# Patient-reported outcomes in multiple sclerosis: a prospective registry cohort study

**DOI:** 10.1093/braincomms/fcad199

**Published:** 2023-08-20

**Authors:** Annalaura Lerede, Jeff Rodgers, Rod M Middleton, Adam Hampshire, Richard Nicholas, The UK MS Register Research Group, Alasdair Coles, Jeremy Chataway, Martin Duddy, Hedley Emsley, Helen Ford, Leonora Fisniku, Ian Galea, Timothy Harrower, Jeremy Hobart, Huseyin Huseyin, Christopher M Kipps, Monica Marta, Gavin V McDonnell, Brendan McLean, Owen R Pearson, David Rog, Klaus Schmierer, Basil Sharrack, Agne Straukiene, David V Ford

**Affiliations:** Department of Brain Sciences, Imperial College London, London W120BZ, UK; Population Data Science, Swansea University Medical School, Swansea SA2 8PP, UK; Population Data Science, Swansea University Medical School, Swansea SA2 8PP, UK; Department of Brain Sciences, Imperial College London, London W120BZ, UK; Department of Brain Sciences, Imperial College London, London W120BZ, UK; Population Data Science, Swansea University Medical School, Swansea SA2 8PP, UK

**Keywords:** multiple sclerosis, patient-reported outcomes, online registry

## Abstract

Registries have the potential to tackle some of the current limitations in determining the long-term impact of multiple sclerosis. Online assessments using patient-reported outcomes can streamline follow-up enabling large-scale, long-term, cost-effective, home-based, and patient-focused data collection. However, registry data are sparsely sampled and the sensitivity of patient-reported outcomes relative to clinician-reported scales is unknown, making it hard to fully leverage their unique scope and scale to derive insights. This retrospective and prospective cohort study over 11 years involved 15 976 patients with multiple sclerosis from the United Kingdom Multiples Sclerosis Register. Primary outcomes were changes in two patient-reported outcomes: Multiple Sclerosis Impact Scale motor component, and Multiple Sclerosis Walking Scale. First, we investigated their validity in measuring the impact of physical disability in multiple sclerosis, by looking at their sensitivity to disease subtype and duration. We grouped the available records (91 351 for Multiple Sclerosis Impact Scale motor and 68 092 for Multiple Sclerosis Walking Scale) by these two factors, and statistically compared the resulting groups using a novel approach based on Monte Carlo permutation analysis that was designed to cope with the intrinsic sparsity of registry data. Next, we used the patient-reported outcomes to draw novel insights into the developmental time course of subtypes; in particular, the period preceding the transition from relapsing to progressive forms. We report a robust main effect of disease subtype on the patient-reported outcomes and interactions of disease subtype with duration (all *P* < 0.0001). Specifically, patient-reported outcomes worsen with disease duration for all subtypes (all *P* < 0.0001) apart from benign multiple sclerosis (Multiple Sclerosis Impact Scale motor: *P* = 0.796; Multiple Sclerosis Walking Scale: *P* = 0.983). Furthermore, the patient-reported outcomes of each subtype are statistically different from those of the other subtypes at all time bins (Multiple Sclerosis Impact Scale motor: all *P* < 0.05; Multiple Sclerosis Walking Scale: all *P* < 0.01) except when comparing relapsing-remitting multiple sclerosis with benign multiple sclerosis and primary progressive multiple sclerosis with secondary progressive multiple sclerosis. Notably, there were statistically significant differences between relapsing-remitting and progressive subtypes at disease onset. Critically, the patient-reported outcomes are sensitive to future transitions to progressive subtypes, with individuals who transition presenting with higher patient-reported outcomes in their relapsing-remitting phase compared to individuals who don’t transition since onset (all *P* < 0.0001). Patient-reported outcomes capture different patterns of physical worsening over disease length and across subtypes; therefore, they are a valid tool to measure the physical impact of multiple sclerosis over the long-term and cost-effectively. Furthermore, more advanced physical disability manifests years before clinical detection of progressive subtypes, adding evidence to the presence of a multiple sclerosis prodrome.

## Teaser

Lerede *et al*. found patient-reported outcomes to be a useful tool for tracking multiple sclerosis progression across subtypes and identifying early disability differences. They used permutation-based methods to analyse patient registry data, demonstrating the potential of online monitoring technologies to estimate multiple sclerosis progression risk early and support clinical decision-making.

## Graphical Abstract

**Graphical abstract:**
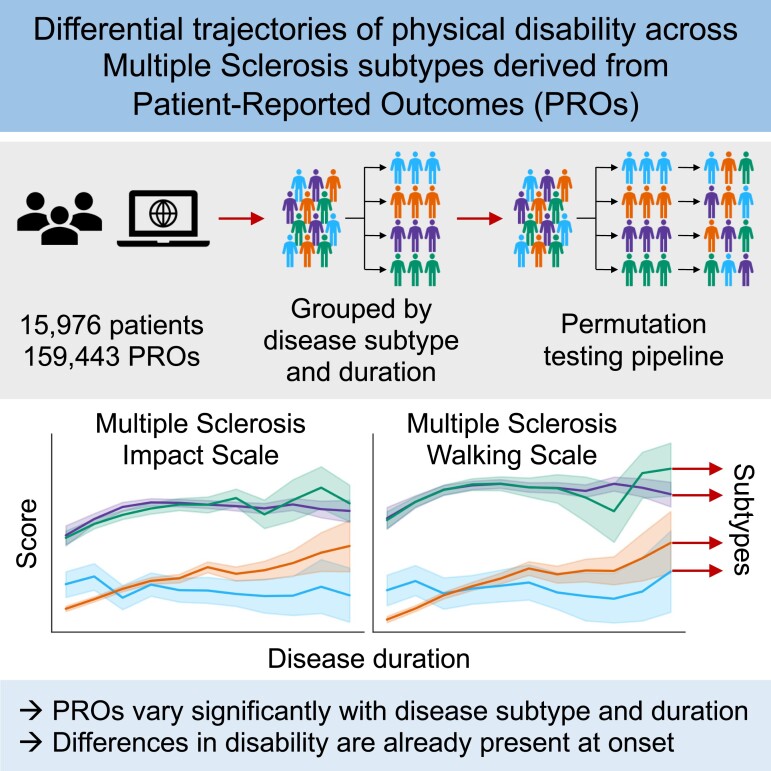

## Introduction

In chronic conditions such as multiple sclerosis, the limitations of randomized controlled trials have been highlighted by their short length, strict entry criteria, and limited ability to determine comparative effectiveness. This is compounded by an emerging view that early treatment is the optimal strategy to maximize patients’ long-term benefits.^1^ This need for prolonged follow-up means that registries are becoming the only viable way of determining the long-term impact of treatments. Registries collect ‘real-life’ data, without a directed research intervention but can be retrospectively analysed to compare outcomes based on a particular exposure. Successful registries at a national^2^ and international level^3^ have delivered key insights focusing predominantly on long-term disability, a feature missing from randomized controlled trials. Traditional registries have in turn been extended by online registries that have widened the range and increased the scale and frequency of data that can be collected including patient-reported outcomes (PROs)^4^ and technologically driven outcomes.^5^ Critically, online registries offer the opportunity for cost-effective home-based longer-term follow-up; this is of particular interest in multiple sclerosis, a disease with a variable outcome, which evolves for some over the short-term but for the majority over the long-term. Furthermore, their usefulness is highlighted by the recent COVID-19 pandemic where the risks to those with disabilities and on immunosuppressive disease modifying therapies attending hospital visits favoured online assessment.^6^

Registries capture what can be described as ‘sparse’ data, as information is not collected regularly at equidistance timepoints, like in a clinical trial, nor it is completed every time by every participant. Data are usually collected in routine clinical practice, most commonly face-to-face, and submitted periodically on an obligatory or voluntary basis. This can result in attrition bias arising due to differences in follow-up and care patterns and detection bias where there are variations in the density and duration of follow-up between centres. Finally, informed censoring occurs when subjects drop out due to surrogate results.^3,7,8^ A further issue, unique to online multiple sclerosis registries, is that the most common outcome used and favoured by regulators, the expanded disability status scale (EDSS), requires face-to-face contact.^9,10^ The EDSS is an ordinal scale based on a neurological examination at lower scores, measured walking distance in the middle range, and finally on function at high scores.^11^ The EDSS has several problems that complicate its use but ironically its major issue is the variability that arises from the requirement to be performed by a trained examiner. This issue is exacerbated in large longitudinal datasets where many examiners are involved who may not follow a standardized protocol as required in trials. Telephone, online and PRO versions have been developed but have differing characteristics to the classic version.^12–14^ Alternative outcome measures have been the focus of much work with regulators.^15,16^ PROs provide one such alternative that aims to collect data entered by patients capturing a range of multiple sclerosis impacts. PROs are potentially a useful approach for registries as they ensure data are entered for the same person by the same person over the longer-term and can be completed conveniently over the internet. The use of PROs as disease biomarkers has been long debated.^17^ For adoption by the wider community measures not only need internal and external validity but also must reliably quantify change over time, e.g. capture how disability is modulated by age and disease duration, differentiate among disease subtypes, and ideally, anticipate progression between subtypes.^16,18^

The United Kingdom Multiple Sclerosis Register (UKMSR) is a registry initiative focused on multiple sclerosis that has been active since 2011 in the UK.^19^ The UKMSR serves as a way for multiple sclerosis patients to connect with one another, report their personal views on their disease and engage with research. It consists of an internet portal where people with multiple sclerosis (pwMS) can take part independent of their healthcare team but also engages with 46 National Health Service (NHS) multiple sclerosis centres where pwMS are recruited and their healthcare professional and objective outcomes can be recorded in an independent and partially overlapping dataset. This has resulted in a unique database, both in terms of size and longitudinal follow-up time. The UKMSR captures a wide range of disease and non-related information including demographics of the users, characteristics of the disease at onset, symptomatology at onset and throughout the disease, and PROs resulting from the periodic administration of online questionnaires.

Here we analysed large-scale register data for two PROs that have been mainstays of the UKMSR—the Multiple Sclerosis Impact Scale (MSIS-29) motor component^20^ and the Multiple Sclerosis Walking Scale (MSWS-12)^21^—both focused on physical disability. These PROs were developed to capture the motor element of the EDSS. First, we developed a simple but flexible statistical approach, based on robust permutation modelling, to maximize the proportion of available data utilized in the context of sparsity. Then, we validated the measures with respect to their sensitivity to expected changes in disease duration and subtype. Finally, we explored the potential of these uniquely large and longitudinal data provided by a register to derive new insights into the nature of change across time, including patterns in the PRO-measured motor deficits that precede the development of progressive multiple sclerosis.

## Materials and methods

### Data collection

The UKMSR is an online, UK-wide register supported by NHS clinical centres (Research Ethics Committee: South-West Central Bristol National Research Ethics Service initially as 16/SW/0194, now 21/SW/0085). The register includes independent verification of treatments and EDSS outcomes from NHS centres in a separate but overlapping population. The register also gathers PROs in the form of questionnaires administered periodically to the users regarding their physical and psychological wellbeing as well as disease-specific information such as about relapses, treatments, etc. Participants entered data 3-monthly from 2011 to 2018 and 6-monthly subsequently after receiving email reminders. Since September 2018 participants have a 28-day window in which to complete the PROs, although often they are all completed in 1 day. Demographic data collected include age, gender, and year of disease onset.

### Patient-reported outcomes

We analysed two of the collected PROs: the MSIS-29 motor and the MSWS-12. The MSIS-29 motor component version 1 (MSIS-29v1)^20^ was used before April 2012, and version 2 (MSIS-29v2)^22^ was used subsequently. Answers to the 20 questions that form the MSIS-29 motor component are each scored between 1 and 5 in version 1 and between 1 and 4 in version 2. As a consequence of this, the scores for MSIS-29v1 give a total ranging from 20 to 100, whereas those for MSIS-29v2 give a total from 20 to 80. To account for the changes in scales, and leverage all data available, the totals for both versions were rescaled to a value in the range of 0–100 using a min-max normalization procedure fitted separately for each version. MSWS-12 version 2 was used to assess walking function.^21^ The score was normalized as above. Participants were excluded from the MSWS-12 assessment if they indicated that they could not walk (the questions weren’t relevant to them).

### Data curation and handling sparsity

Online self-reported data are by nature large but also sparse; therefore, we did not have complete demographic and clinical records for everyone, nor did the users complete the available PROs at every collection window. We applied a conservative approach to this sparsity, keeping as many observations as possible for each analysis. The minimum information needed to be included in the study was to have completed at least one of the considered PROs once, to have recorded a legitimate year of birth and symptoms onset and to have a multiple sclerosis subtype at completion date known or derivable, as detailed below. The pre-processing, visualization, and analysis steps included: (i) assignment of multiple sclerosis subtype labels at individual timepoints; (ii) pooling the observations into disease length groups; (iii) plotting the PRO-derived trajectories over disease length stratified by disease subtype and transitioning subtype; (iv) plotting the PROs’ distributions at each disease time bin stratified by subtype; (v) evaluating the presence of a main statistical effect of disease duration and subtype on the scores as well as their interaction; (vi) when a main effect was detected, performing *post hoc* pairwise comparisons to understand which groups were statistically distinct and in what direction.

### Assignment of multiple sclerosis subtype labels at patient-reported outcome timepoints

Disease subtype can change with disease progression and in some cases can only be established over time. For this reason, users of the register are asked every 18 months to update their subtype based on what they have been told by their clinical team. Nonetheless, the dates when these updates are made do not always coincide with the dates when users complete the PROs. Consequently, it was necessary to determine the subtype at any given PRO timepoint according to the time course of recorded classifications for each user (Supplementary Fig. 1).

Specifically, there are four main multiple sclerosis subtypes: relapsing-remitting (RRMS), secondary progressive (SPMS), primary progressive (PPMS) and benign (BN). At first diagnosis, pwMS are labelled as RRMS, SPMS, PPMS, or do not know. If there are no further episodes, then patients are subsequently classified as BN (it takes at least 15 years from onset to confirm a BN label).^23^ People with RRMS may transition to SPMS (the estimated median time for transitions to happen is about 19 years from onset).^24^

For analysis, class labels at any given PRO timepoint were determined based on the time course of subtype answers in the following manner: (i) People with do not know classifications early in the time course had these classifications replaced with the first subsequent valid label. (ii) People who were classified later in the time course as either PPMS or BN had these labels applied to all preceding timepoints retrospectively, as by definition neither subtype can transition to another. (iii) People with SPMS labels at baseline were assumed to remain SPMS for all subsequent timepoints as this subtype cannot progress to another. (iv) People who were designated as RRMS at all timepoints were labelled as such. (v) Timepoints for people who were designated as RRMS at baseline but who transitioned to SPMS were assigned the temporally closest recorded label. Timepoints where the offset between the questionnaire completion date and the label registration was greater than 2 years were excluded. This cut-off was chosen because transitions occur over time, and it can take over 1 year to confirm a transition, for example from RRMS to SPMS. Follow-up was for a maximum of 11 years. Consequently, observations labelled as BN and included between 0 and 10 years from the onset were so based on a maximum of 11 years follow-up and are indicated with a dotted line in the plots.

**Figure 1:**
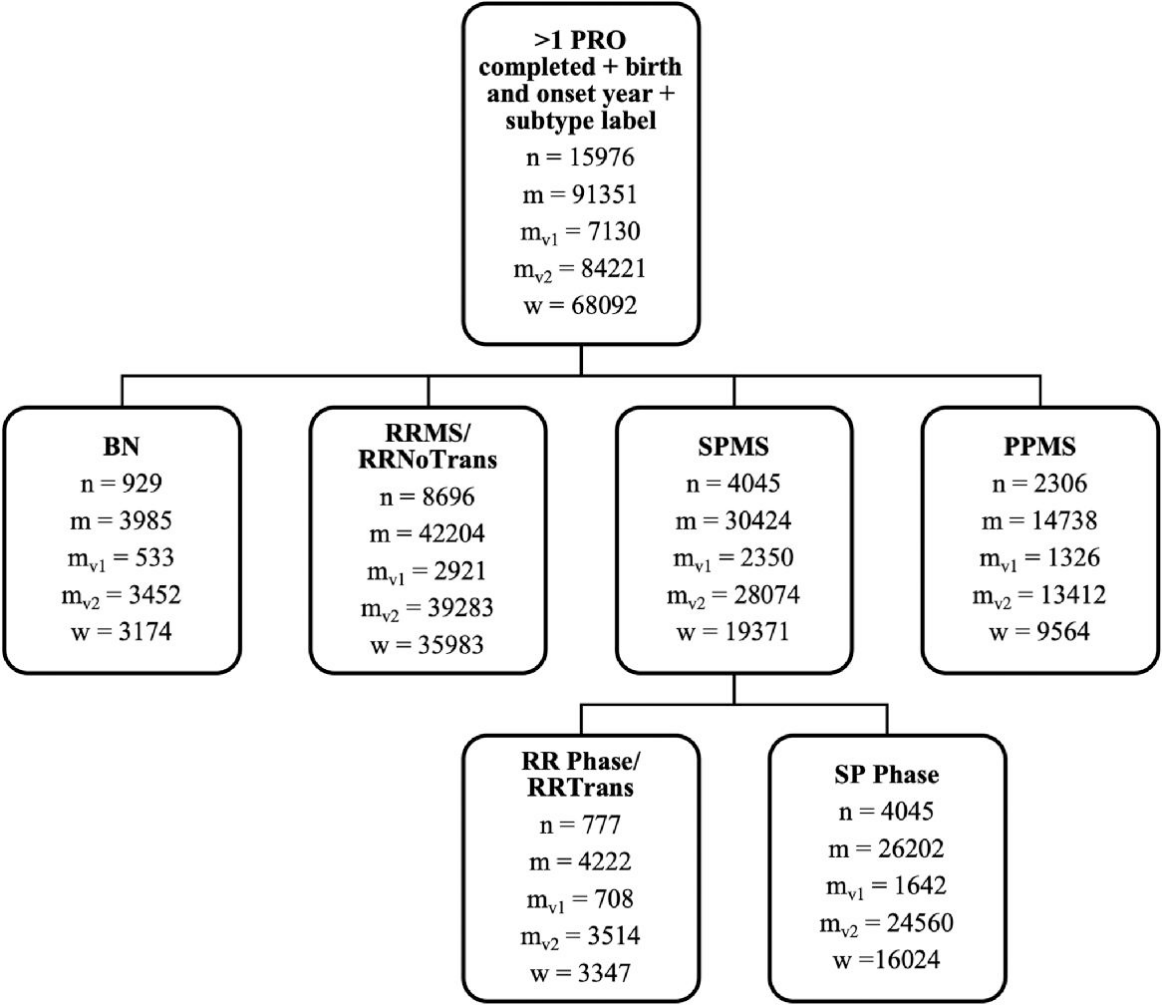
**Flowchart illustrating how the studied subgroups are derived from the overall UKMSR population.** For each subgroup we reported the number of unique users (**n**) and the total number of MSIS-29 motor (**m**) and MSWS-12 (**w**) questionnaires completed by the users. We also reported how many of the MSIS-29 motor questionnaires completed were respectively version 1 (*m_v_*_1_) and version 2 (*m_v_*_2_) of the questionnaire. The minimal requirements to be included in the study consist of having completed at least one PRO, having reported a legitimate year of birth and symptoms onset and having a disease subtype label at completion date known or derivable.

Of particular interest is the question of whether there are differences early in the time course of multiple sclerosis for RRMS people who do versus do not subsequently transition to SPMS. For the first part of the analyses, we grouped the PROs of individuals who transitioned before or during our follow-up together, regardless of whether they were collected during the individuals’ relapsing-remitting (RR) or secondary progressive (SP) phase. We gave these observations the label SPMS retrospectively. Consequently, we analysed and compared records across four subtypes, namely: BN, RRMS, SPMS and PPMS. In the second part of the analyses instead, we only considered PRO timepoints in the RR phase and we divided them based on whether the respective patients transitioned or not during the follow-up time. In this way we derived two transitioning subtypes: RRNoTrans (coincides with RRMS from above) and RRTrans (a subset of SPMS from above). A visual explanation of the nested grouping is reported in Supplementary Fig. 1. The number of unique users, and the total number of MSIS-29 and MSWS-12 records for each group is reported in Fig. 1.

### Patient-reported outcomes-derived trajectories with disease duration

The evolution of the mean PRO-derived scores for the different subtypes was examined as a function of disease duration. Observations were grouped into 5-year blocks starting from 0 to 50 years with disease. Disease duration was defined as the time from the first symptom onset to the time of PRO observation. Observations at disease duration >50 years were formed into one further group due to low frequency.

To prevent repeated observations within time bins, we averaged all observations from a single individual within a time bin. This allowed us to leverage all available data towards more representative measurements within each timeframe, whilst meeting the assumption of group independence, needed for subsequent analyses.

### Statistical analysis

Data curation, pre-processing, and statistical analyses were conducted in Python 3.7.3. Although UKMSR PRO data are powerful in terms of population size and timescale, they present challenges for statistical analysis; specifically, (i) groups’ sizes are imbalanced (both in terms of disease time bins and subtypes), (ii) data are sparse, (iii) the number of observations varies across individuals, and (iv) distributions of scores are non-Gaussian. A robust pipeline based on Monte Carlo permutation analysis was developed to address these challenges. Permutation analysis is a natural choice because it quantifies the probability of the observed cross-condition or cross-participant differences given random permutations of the exact data, thereby coping with imbalanced class sizes and non-normally distributed data whilst enabling complexities, such as the variable number of observations per subject, to be controlled for when generating the null distribution.^25,26^

Steps applied in the pipeline were as follows (and are also illustrated in Supplementary Fig. 2). (i) Dependent on whether group size was two groups or multiple groups/factors, the real observed across groups t or F statistic was calculated from the non-permuted data (*t*_real_ or *F*_real_, respectively). (ii) Data labels were permuted across observations with randomization constrained to account for potentially confounding factors (i.e. by switching labels across subjects with the same number of observations thereby preserving the level of engagement). (iii) The t or F value was recalculated for the permuted data (*t*_perm_ and *F*_perm_). (iv) Steps ii–iii were repeated 10 000 times, producing the null distribution. (v) *P* values were calculated according to where the real observed statistic ranked within the permutation null distribution.

When a main effect was detected, and if more than two groups were being compared, pairwise permutation analyses were performed to further characterize the basis of the observed main effect. Unless otherwise stated, a two-tailed alpha threshold of *P* < 0.05 was applied for rejecting the null hypothesis.

Specifics of individual permutation analyses are detailed in the results section and population sample sizes per analysis are reported in Fig. 1 and Table 1. Plots derived from the permutation testing, showing the null distribution of permuted statistics as well as the real statistic, can be found in Supplementary Figs 3–6.

**Table 1:** Characterization of PRO responses of the UKMSR population with the minimal requirements at all time points

Disease length (yrs)	Total *N*	Disease subtype					Mean age (yrs, range)	Gender (female, missing)
		BN	RRMS-RRNoTrans	SPMS		PPMS		
				RR Phase-RRTrans	SP Phase			
0–5	9339/7934	265/219	7288/6314	294/224	306/259	1186/918	42.2, 18–82	7477, 3
5–10	16 703/13 772	401/319	10 818/9329	724/557	1570/1124	3190/2443	47.4, 18–84	12987, 29
10–15	16 272/12 603	497/388	8807/7557	696/565	2940/1954	3332/2139	50.8, 19–86	12296, 10
15–20	13 839/10 227	610/501	6094/5216	702/546	3797/2289	2636/1675	53.7, 25–84	10398, 17
20–25	11 187/7747	539/423	3906/3263	583/463	4339/2593	1820/1005	55.8, 26–86	8386, 9
25–30	8660/5858	504/410	2309/1883	523/433	4170/2464	1154/668	58.1, 32–87	6575, 3
30–35	6322/4072	443/354	1403/1128	319/261	3553/2038	604/291	60.3, 37–86	4983, 0
35–40	4077/2685	255/203	812/663	179/131	2477/1515	354/173	62.6, 43–91	3095, 1
40–45	2639/1714	223/167	493/409	94/70	1602/938	227/130	65.3, 46–88	1938, 0
45–50	1393/941	141/118	180/148	72/68	835/507	165/100	68.0, 51–84	1088, 0
>50	920/539	107/72	94/73	36/29	613/343	70/22	70.9, 58–91	737, 0

MSIS-29/MSWS-12 records counts are reported.

## Results

### Descriptive analysis of user engagement with the United Kingdom Multiple Sclerosis Register

**Figure 2:**
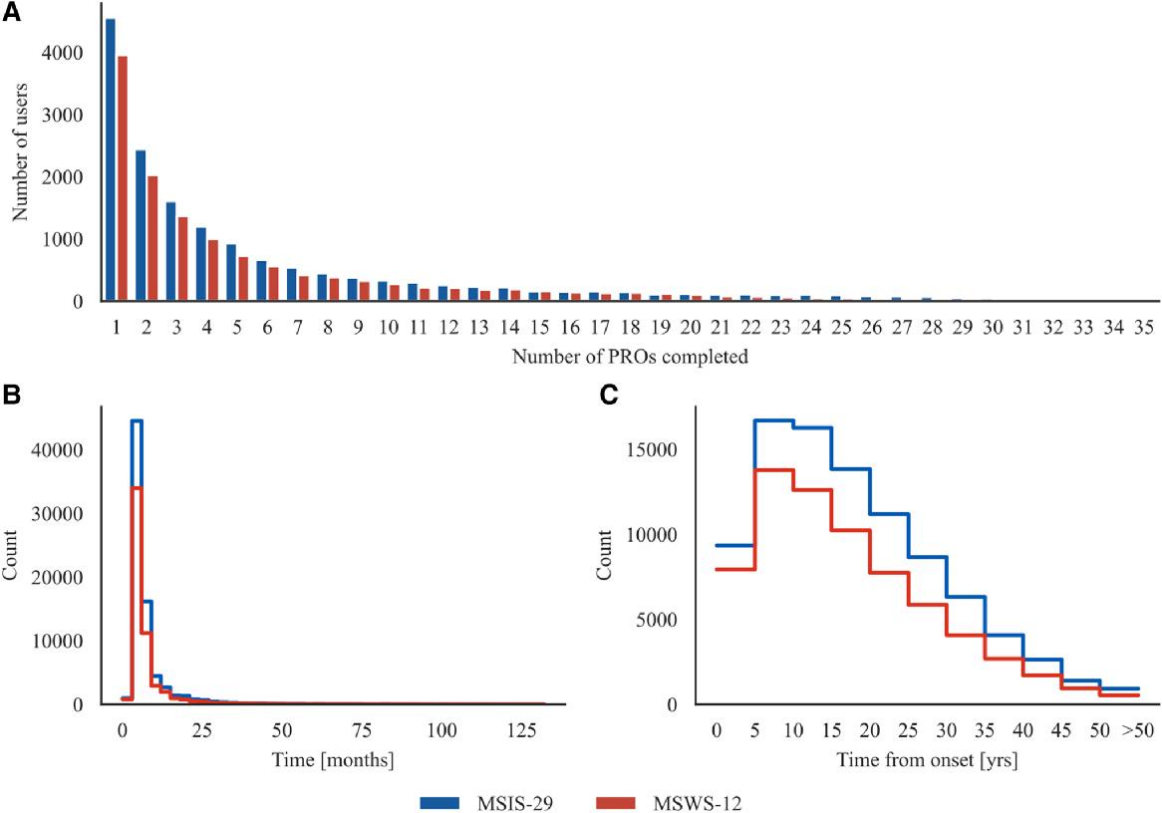
**Analysis of user engagement with the UKMSR.** (**A**) Frequency of users who completed a specific number of times MSIS-29 motor and MSWS-12. 62% of the users for MSIS-29 motor and 64% for MSWS-12 completed between 1 and 4 PROs. (**B**) Distribution of the time intervals elapsed between consecutive completions of MSIS-29 motor (time_mean_ = 7 months, time_median_ = 5 months, time_max_ = 131 months) and MSWS-12 (time_mean_ = 7 months, time_median_ = 4 months, time_max_ = 113 months) across all users. **(C**) Number of times MSIS-29 motor and MSWS-12 were completed at the different disease time bins.

Data were collected over 132 months, from May 2011 to April 2022. Users joined the UKMSR in a rolling manner at different points in time and in the disease. They could choose to engage with any of the PROs available during the collection windows. As a result, users completed the PROs a different number of times, at a variety of timepoints over the collection period, with 62% of the users for MSIS-29 motor and 64% for MSWS-12 having completed between 1 and 4 questionnaires thus far (Fig. 2A). The distribution of the time intervals elapsed between subsequent questionnaire completion dates across all users is displayed in Fig. 2B and indicates how often users engage with the register (MSIS-29 motor: time_mean_ = 7 months, time_median_ = 5 months, time_max_ = 131 months; MSWS-12: time_mean_ = 7 months, time_median_ = 4 months, time_max_ = 113 months). This shows how, even though many of the users that completed the PROs in a certain collection window also completed them in the previous window (active users), others come back to engage with the UKMSR after a gap of many years. The number of MSIS-29 motor and MSWS-12 collected at different disease time bins is reported in Fig. 2C. The flowchart in Fig. 1 illustrates how each of the studied subgroups was derived from the overall UKMSR population. The characterization of PRO responses at all timepoints is reported in Table 1; the users are accounted for as many times as the number of questionnaires they have completed at different timepoints.

### Validity of the patient-reported outcomes

We first assessed whether the considered PROs are valid measures of physical disability in multiple sclerosis, looking for statistical differences in the scores as a function of disease subtype and disease duration. Given that multiple sclerosis is a disease with a highly variable outcome dependent on subtype that plays out mostly over the long-term, we hypothesized that a good marker of physical disability for this disease should be sensitive to both subtype and duration.

#### Patient-reported outcomes evolve differently across subtypes

**Figure 3:**
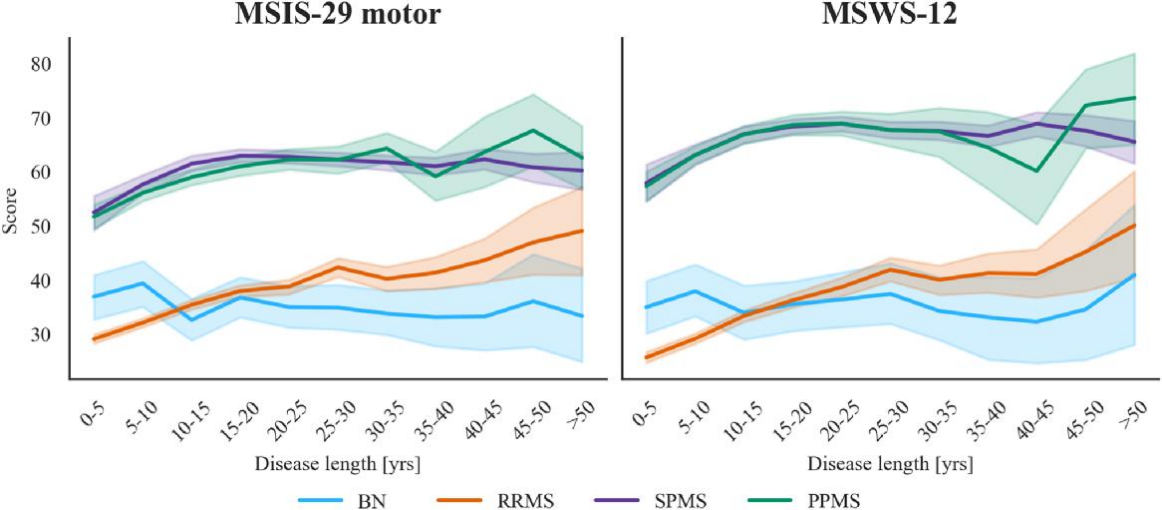
**Mean score trajectories with 95% confidence intervals (CI) over disease length for the different disease subtypes for MSIS-29 motor (left panel: BN = 3985, RRMS = 42204, SPMS = 30424, PPMS = 14 739) and MSWS-12 (right panel: BN = 3174, RRMS = 35983, SPMS = 19371, PPMS = 9564).** Performing permutational multivariate analysis of variance (*n* = 10 000 permutations), a robust main effect of disease subtype was found on the scores (MSIS-29 motor: *F*_real_ = 2698, *P* < 0.0001; MSWS-12: *F*_real_ = 3007, *P* < 0.0001) as well as an interaction of disease subtype and duration (MSIS-29 motor: *F*_real_ = 3.9, *P* < 0.0001; MSWS-12: *F*_real_ = 3.5, *P* < 0.0001).

First, we examined differences in PROs as a function of disease length by disease subtype. To visualize this, we plotted the mean score trajectories with 95% confidence intervals of the two PROs over disease duration for the different subtypes (Fig. 3). This was done by pooling observations together in 5-year bins starting at onset separately for each subtype and evaluating the mean score for each bin. We obtained the trajectories from 91 351 observations for MSIS-29 motor and 68 092 for MSWS-12. Figure 3 shows differences in trajectories by subtype from onset through the full disease course. In particular, BN and RRMS have lower average scores compared to PPMS and SPMS, since onset and throughout.

Permutation testing was performed by randomly reassigning 10 000 times subtype labels while preserving any differences in the level of engagement per subtype when estimating the null distribution. The F statistic was evaluated for the real and permuted data. We found a robust main effect of disease subtype on the scores (MSIS-29 motor: *F*_real_ = 2698, *P* < 0.0001; MSWS-12: *F*_real_ = 3007, *P* < 0.0001) and an interaction of disease subtype and duration (MSIS-29 motor: *F*_real_ = 3.9, *P* < 0.0001; MSWS-12: *F*_real_ = 3.5, *P* < 0.0001).

#### Disease length influences all subtypes but benign

Given that an interaction between disease length and subtype was detected, we next looked for statistical differences in PROs as a function of disease length, separately for each subtype. Permutation testing was performed randomly reassigning 10 000 times disease time bins for each subtype while preserving the level of engagement. We found that disease length had an association with the scores for RRMS (MSIS-29 motor: *F*_real_ = 41.4, *P* < 0.0001; MSWS-12: *F*_real_ = 46.1, *P* < 0.0001), PPMS (MSIS-29 motor: *F*_real_ = 10.0, *P* < 0.0001; MSWS-12: *F*_real_ = 8.3, *P* < 0.0001), and SPMS (MSIS-29 motor: *F*_real_ = 7.0, *P* < 0.0001; MSWS-12: *F*_real_ = 6.5, *P* < 0.0001), but not for BN (MSIS-29 motor: *F*_real_ = 0.86, *P* = 0.796; MSWS-12: *F*_real_ = 0.37, *P* = 0.983). This makes intuitive sense as BN individuals have a milder form of multiple sclerosis and experience less, if any, physical disability. We can visually inspect this in Fig. 3 where the BN trajectory fluctuates around the same range of values, while the other trajectories weakly (SPMS: MSIS-29 *m* = 0.31, MSWS-12 *m* = 0.46; PPMS: MSIS-29 *m* = 1.50, MSWS-12 *m* = 1.28 slope coefficients obtained fitting a line through the data separately for each subtype) or strongly (RRMS: MSIS-29 *m* = 2.11, MSWS-12 *m* = 2.65) increase over disease time. The SPMS trajectory is characterized by a lower gradient coefficient when fitting a line through the data among the subtypes that show an association with disease length. This can be attributed to the fact that SPMS individuals show an increase in their scores for the first 20 years with disease but appear to plateau afterwards.

#### Patient-reported outcomes are sensitive to disease subtype within all disease time bins

**Figure 4:**
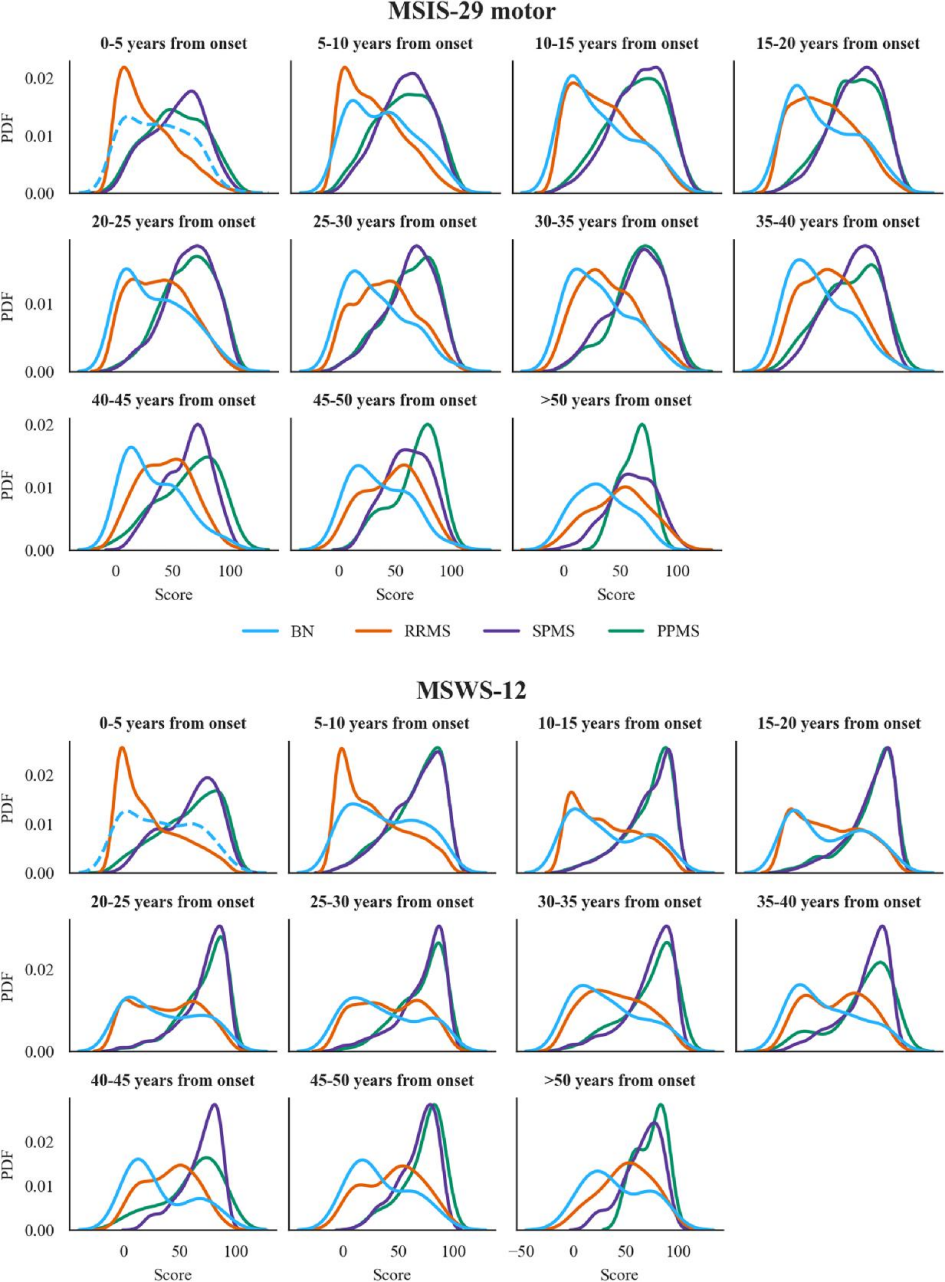
**MSIS-29 motor (top panel) and MSWS-12 (bottom panel) probability density functions stratified by subtype for the different disease time bins considered.** The probability density curve for the BN subgroup between 0 and 5 years from the onset is dotted as this label was given retrospectively and was based on a maximum of 11-year follow-up. Performing permutational multivariate analysis of variance (*n* = 10 000 permutations), a robust main effect of disease subtype was found on the scores at disease onset and throughout all time bins considered (MSIS-29 motor: all *P* < 0.0001; MSWS-12: all *P* < 0.0001). The F statistics across all time bins are reported in Supplementary Table 2.

Motivated by these findings, we tested whether PROs differed across subtypes within all disease time bins. First, we plotted the probability density functions of the PROs (Fig. 4) stratified by disease subtype for each time bin. The mean, standard deviation and skew of the distributions are reported in Supplementary Table 1. SPMS and PPMS presented similar distributions and centred around higher values compared to RRMS and BN at all intervals. Relapsing and progressive subtypes had distinct patterns at each disease length with progressive distributions consistently being left-skewed and relapsing distribution mostly presenting with a right-skew. This pattern was more evident for MSWS-12. RRMS distributions were similar to BN distributions early on in the disease but with increasing time from onset, they departed from these, losing the positive skew, shifting towards higher values and approaching the progressive distributions (*skew* goes from 0.8 to −0.1 over 50 years with disease for MSIS-29 and from 0.8 to −0.3 for MSWS-12). Overall, the mean of the RRMS distribution was the one that changed the most with increasing disease length (*mean* from 29 to 49 for MSIS-29 motor and from 26 to 50 for MSWS-12). This makes sense intuitively as the RRMS group comprises individuals who have not reported a transition to SPMS during the current follow-up but might still transition later in the disease. These transitions, which occur gradually over time, were represented in the plots as the loss of positive skew of the RRMS distribution. On the contrary, the BN distribution remained right-skewed (*skew* always above 0.2). Permutation testing by randomly reassigning 10 000 times disease subtype labels separately for each time bin showed a robust association of PROs with disease subtype at disease onset and throughout the time bins (MSIS-29 motor: all *P* < 0.0001; MSWS-12: all *P* < 0.0001). A complete record of F statistics and *P* values can be found in Supplementary Table 2.

#### Pairwise comparisons

Finally, we performed *post hoc* pairwise comparisons to investigate which subtypes differed from each other. We randomly permuted subtype labels 10 000 times in pairs, first overall while preserving the level of engagement, and then separately within each disease time bin. We found that overall, the scores of all subtypes were different from the others (MSIS-29 motor: all *P* < 0.0001; MSWS-12: all *P* < 0.05) except when comparing the MSIS-29 scores of BN and RRMS individuals (*P* = 0.51). Regarding pairwise comparisons within each time bin, respectively, 91% and 41% of the *P* values were not significant when comparing SPMS with PPMS or RRMS with BN (complete t statistics and *P* values are reported in Supplementary Table 3). This can be visually validated in Fig. 4 where the distributions of SPMS and PPMS appear similar in most of the time bins. The same held true for the distributions of BN and RRMS, especially early on. The scores for all the other subtypes’ combinations were, instead, significantly different at all time bins (MSIS-29 motor: at least *P* < 0.05; MSWS-12: at least *P* < 0.01).

### Deriving new insights using the patient-reported outcomes

**Figure 5:**
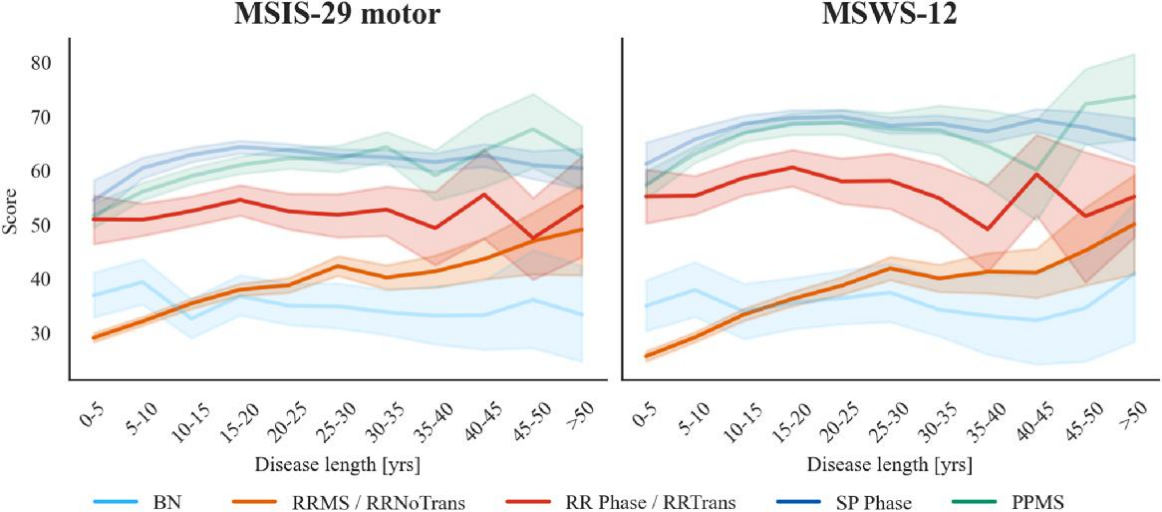
**Mean score trajectories with 95% CI over disease length for transitioning (RRTrans: MSIS-29 = 4222, MSWS-12 = 3347) and non-transitioning (RRNoTrans: MSIS-29 = 42202, MSWS-12 = 35 983) individuals in our cohort taken in their RR phase for MSIS-29 motor (left panel) and MSWS-12 (right panel).** Being RRTrans was associated with higher scores on average in both MSIS-29 motor (*t*_real_ = −23.5, *P* < 0.0001) and MSWS-12 (*t*_real_ = −26.2, *P* < 0.0001) when performing permutation testing with t statistic and *n* = 10 000 permutations. Mean score trajectories with 95% CI for BN (MSIS-29 = 3985, MSWS-12 = 3174), PPMS (MSIS-29 = 14738, MSWS-12 = 9564) and SPMS individuals in their SP phase (MSIS-29 = 30424, MSWS-12 = 19 371) are also displayed here in transparency for comparison and were not included in the statistical tests.

In the previous sections, we advocated the validity of the PROs as measures of physical disability in multiple sclerosis as they proved to be sensitive to the diverse levels of physical impairment that people belonging to different subtypes experience in the various stages of the disease. Motivated by these findings, we investigated further the utility of this type of data in providing novel insights into the developmental time course of disease subtypes. A pressing matter in multiple sclerosis involves defining among the relapsing individuals those who are going to become progressive, and ideally being able to do so early on, to treat patients accordingly. Since our *post hoc* pairwise comparisons showed that the PROs for RRMS and SPMS patients are significantly different at every time bin, we extracted from the SPMS group only the observations in the RR phase (RRTrans) and compared those to the RRMS observations (RRNoTrans) to investigate whether the PROs in the RR phase vary as a function of whether the patients will transition or not. Figure 5 shows the mean score trajectories with 95% confidence intervals of the PROs over disease length for the two RR groups, namely RRNoTrans and RRTrans. We obtained the trajectories from 46 426 observations for MSIS-29 motor and 39 330 for MSWS-12. The trajectories of the other subtypes (BN, PPMS and SPMS in the SP phase) are displayed only for comparison. The RRNoTrans and RRTrans trajectories look different since onset and up to around 45 years with disease: RRTrans have on average higher scores compared to RRNoTrans, especially at onset. To validate this, we performed permutation testing randomly reassigning the subtype labels between these two groups 10 000 times both overall, preserving the level of engagement, and separately within each time bin. The t statistic was evaluated for the real and permuted data. We found that the PROs of the two groups are significantly different overall (MSIS-29 motor: *t*_real_ = −23.5, *P* < 0.0001; MSWS-12: *t*_real_ = −26.2, *P* < 0.0001) and at each disease time bin up to 45 years with disease for MSIS-29 motor (at least *P* < 0.05) and to 35 years for MSWS-12 (at least *P* < 0.001) but they become non-significant above this, where notably, data are more sparsely sampled (complete t statistics and *P* values are reported in Supplementary Table 4). This means that the PROs of RR individuals who have transitioned to progressive forms of multiple sclerosis were on average higher since disease onset and subsequently than the PROs of RR individuals who haven’t transitioned during our follow-up timeframe.

## Discussion

Our novel permutation-based analysis approach enables us to handle the complexity and sparsity inherent to registers, thereby retaining the highest proportion of records possible from 11 years of prospective UKMSR PRO data whilst drawing robust statistical inferences regarding the long-term trajectories of decline that should generalize beyond just the most engaged participants. Using this approach, we show that both PROs are viable monitoring tools as they can capture at a large population scale the distinct patterns of physical worsening occurring across different subtypes. In further support of the utility of register-based PROs, our analyses detect subtle differences across multiple sclerosis subtypes early in the disease that anticipate subsequent changes in clinical labels. This highlights the potential of online monitoring technologies to estimate progression risk early and support clinical decision-making.

There are characteristic strengths and weaknesses ubiquitous to registry data that are essential traits of the data collection method. Most notably, the ideal scenario, to have all scales completed by all participants at all collection windows since disease onset, is fundamentally not feasible due to the requirement to repeatedly engage participants remotely, over long timeframes and at large-scale. Consequently, data are inherently sparse and complex with engagement level being a prominent confounder, characteristics that present challenges from the analysis perspective.

Using a permutation-based approach that both handles and explicitly accounts for engagement, enables as much of the available data as possible to be leveraged. Specifically, we included 91% of the total records for MSIS-29 and 93% for MSWS-12, thereby summarising over 91k records for MSIS-29 motor and 68k records for MSWS-12. This produces substantial power enabling us to derive robust statistical conclusions regarding the four trajectories representative of the differential evolution of physical disability across subtypes.

Notably, these trajectories together with the distributions of the scores at different time bins, provide a strong validation of the utility of PROs as large-scale monitoring tools. These plots frame the sensitivity of these PROs to both subtype and duration, as supported by our statistical findings. For example, the distributions for BN individuals’ span lower values compared to those of the other subtypes and their trajectory does not worsen over time. This is expected as BN patients are characterized by minimal physical disability and sporadic if not completely absent relapses.^23^ Conversely, RRMS PROs change the most, presenting with the steepest mean trajectory, and are characterized by a distribution that loses the positive skew as the disease evolves. Again, this pattern of results has clear validity because RRMS patients are periodically affected by relapses, which can become increasingly debilitating, and they can potentially transition to a progressive phenotype, thereby showing a more continuous silent decline.^27^ Consequently, the PROs analysed here provide valid alternatives to established clinical-observational scales such as the EDSS for monitoring physical disability in multiple sclerosis, allowing patients to be monitored at scale, remotely, more often and for longer follow-ups.

Analysis of these PROs at the timescale and population scale of the register produces new insights into the process by which some individuals phenoconvert between subtypes. Indeed, current knowledge in multiple sclerosis supports an individualized approach to treatment with an increasing focus on early intervention to limit later damage.^28^ Here, both of the PROs that we analyse prove sensitive to cross-group differences in patterns of early disability: more specifically, not only do we observe a statistical difference in the earliest time bins between RR and progressive subtypes, we also show a robust statistical difference at those same early timepoints between the PROs of RR individuals who are versus are not going to phenoconvert to the SPMS subtype later in the disease. Therefore, RRMS patients who are at higher risk of becoming progressive may be identifiable early in the disease course. These results are consistent with insights from the pathology and spinal fluid inflammatory profile,^29^ cortical MRI lesion development^30^ and evidence of cognitive dysfunction at presentation.^31^ It is known that early symptoms can be vague and ignored by patients and healthcare professionals, but increasingly there is evidence of a multiple sclerosis prodrome;^32^ as in the presence of early disease symptoms and impacts years before when a diagnosis is made.

The potential strengths in scope and scale of register data have challenges that extend beyond sparsity and complexity.^33^ For example, a related challenge is rooted in the fact that engagement in registries is voluntary, which could result in inferences that generalize poorly to the broader multiple sclerosis population due to sampling bias. While bias is always present when using any sampling method,^34,35^ looking at relative proportions of subtypes in our sample, they appear in close concordance with those known from epidemiological studies.^36,37^ Specifically, the UKMSR sample is representative of the multiple sclerosis population extending prior comparisons^19^ with 14% of the patients being PPMS and 80% being RRMS at disease onset.^36,37^ In turn, consistent with this, overall one-third of RRMS patients transition to SPMS.^38^ Notably, 6% were defined as BN. This is 3% higher than recent estimates.^23^ Overall, while the dataset inevitably has some bias, it appears to be small and is mitigated by our inclusive approach to analysis.

Regarding sampling bias across time, the number of observations collected varies with disease time: reaching a maximum at 5–10 years from onset and then reducing monotonically with disease duration. This may reflect that patients engage less as multiple sclerosis progresses and are more likely to be recruited in early stages. This bias can lead to complications in interpreting certain aspects of the data. For example, it appears that the SPMS PRO trajectories plateau after 15–20 years from onset (Fig. 3). This might indicate that physical disability in our sample stabilizes over time. However, time-dependent sampling bias could also be a contributing factor. If participants that have more severe physical disability to begin with are more likely to drop out, then this would present as a plateau. To test whether this might be the case, we split the SPMS individuals from the 10–20 years from onset groups into those that did and those that did not return in the 20–30 years from onset groups. Then, using a permutation model, we tested for a significant difference between the PROs of these two subgroups. The results showed that SPMS individuals who returned had significantly lower scores on average (mean MSIS-29 motor = 59.3; mean MSWS-12 = 67.3) than those who did not (mean MSIS-29 = 68.1; mean MSWS-12 = 74.1) with *P* values less than 0.0001. While this finding could explain the observed plateau, it does not rule out the possibility that physical disability stabilizes to some degree over time.

In combination with other causes of sampling bias, these results further highlight the challenges of working with register data and the importance of both coping with and accounting for engagement bias when evaluating between-subjects effects during the analysis. Our permutation-based approach achieves this, by permuting the PROs of individuals after matching them by level of engagement, i.e. controlling for it.

Another notable limitation is that the UKMSR currently has a maximum 11-year follow-up since its inception. This means that the individuals who joined the register at onset and are currently labelled as BN could still phenoconvert to more aggressive disease subtypes. This explains why the proportion of BN individuals in our sample is slightly above the population estimate. Similarly, some patients within the RRMS subgroup will transition to SPMS as this happens typically within 15–20 years from onset,^24^ and our follow-up has not allowed enough time for all transitions to happen. This means that the significant cross-group differences observed between multiple sclerosis subtypes are likely to underestimate the true underlying differences in motor impairment trajectories that can be expected as longer follow-up data become available.

Overall, our study supports the value of PRO data and online registry initiatives. Our approach allows us to maximize data usage in a sparse dataset and can be easily applied in the context of other registries and extended to answer different questions. Most importantly, we demonstrate that the more pronounced disability of those who will become progressive is evident early in the natural history of the disease, that is, years before the clinical label is changed. Register-based PROs enable us to detect this gap as they provide data at a larger population scale, across longer timespans and at a more affordable cost than traditional cohort or clinical trial studies. In the future, we intend to determine whether this early disability is also present on mood and sleep scales or when objectively assessing cognitive symptoms. The combination of these findings will allow us to evaluate the potential for assigning individualized progression risk quotients based on the multivariate profile of symptoms at the early stage.

## Supplementary Material

fcad199_Supplementary_Data

## Data Availability

Access to further analyse the data from this study can be provided to accredited researchers following institutional review board approval and completion of appropriate access paperwork (General Data Protection Regulation and safe researcher training). For further information, contact Rod Middleton at r.m.middleton@swansea.ac.uk.

## Appendix 1

The UK MS Register Research Group collaborators: Alasdair Coles, Jeremy Chataway, Martin Duddy, Hedley Emsley, Helen Ford, Leonora Fisniku, Ian Galea, Timothy Harrower, Jeremy Hobart, Huseyin Huseyin, Christopher M Kipps, Monica Marta, Gavin V McDonnell, Brendan McLean, Owen R Pearson, David Rog, Klaus Schmierer, Basil Sharrack, Agne Straukiene, David V Ford.
