# Supplementary material for: Patient-reported outcomes in multiple sclerosis: a prospective registry cohort study
Source: Brain Commun. 2023 Aug 20;5(4):fcad199. doi: 10.1093/braincomms/fcad199 (PMC10440194; doi:10.1093/braincomms/fcad199)
Supplement: fcad199_Supplementary_Data [file fcad199_supplementary_data.docx]

# Supplementary Material

**Supplementary Figure 1 Schematic of disease subtype labels assignment.** Illustration of how multiple sclerosis subtype labels at questionnaire completion dates were derived from the time course of recoded labels for each user and of how the records were subsequently grouped based on these labels.


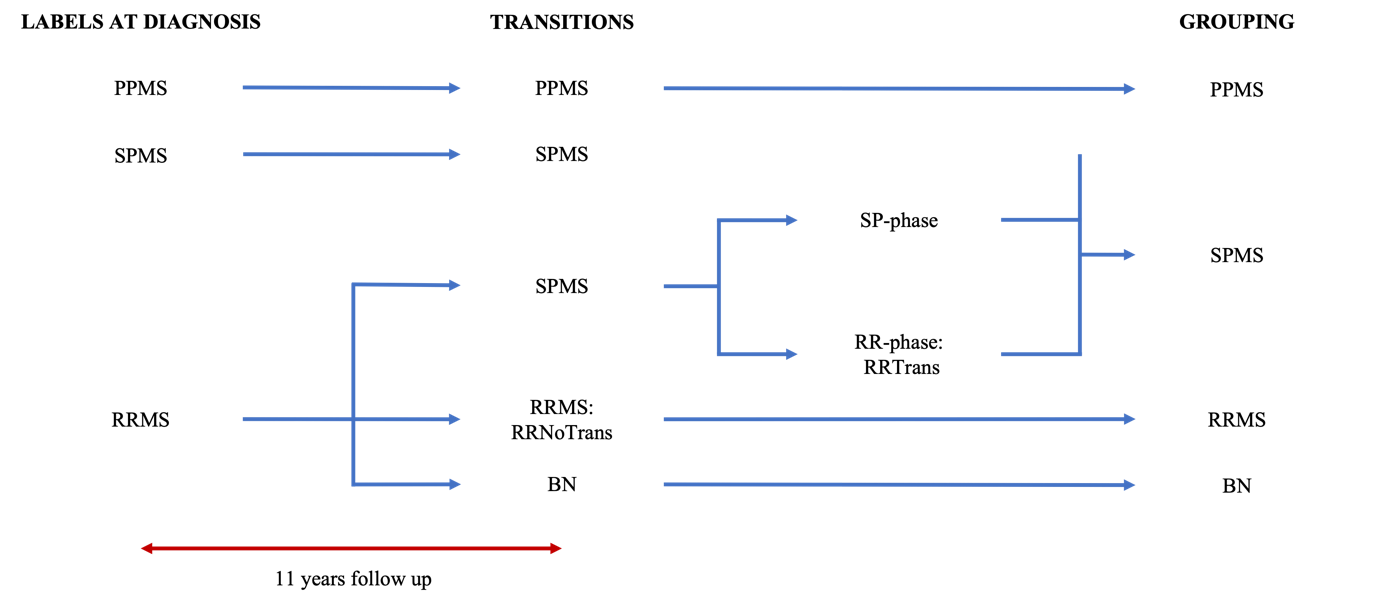


**Supplementary Figure 2 Schematic explaining how permutation testing was carried out.**


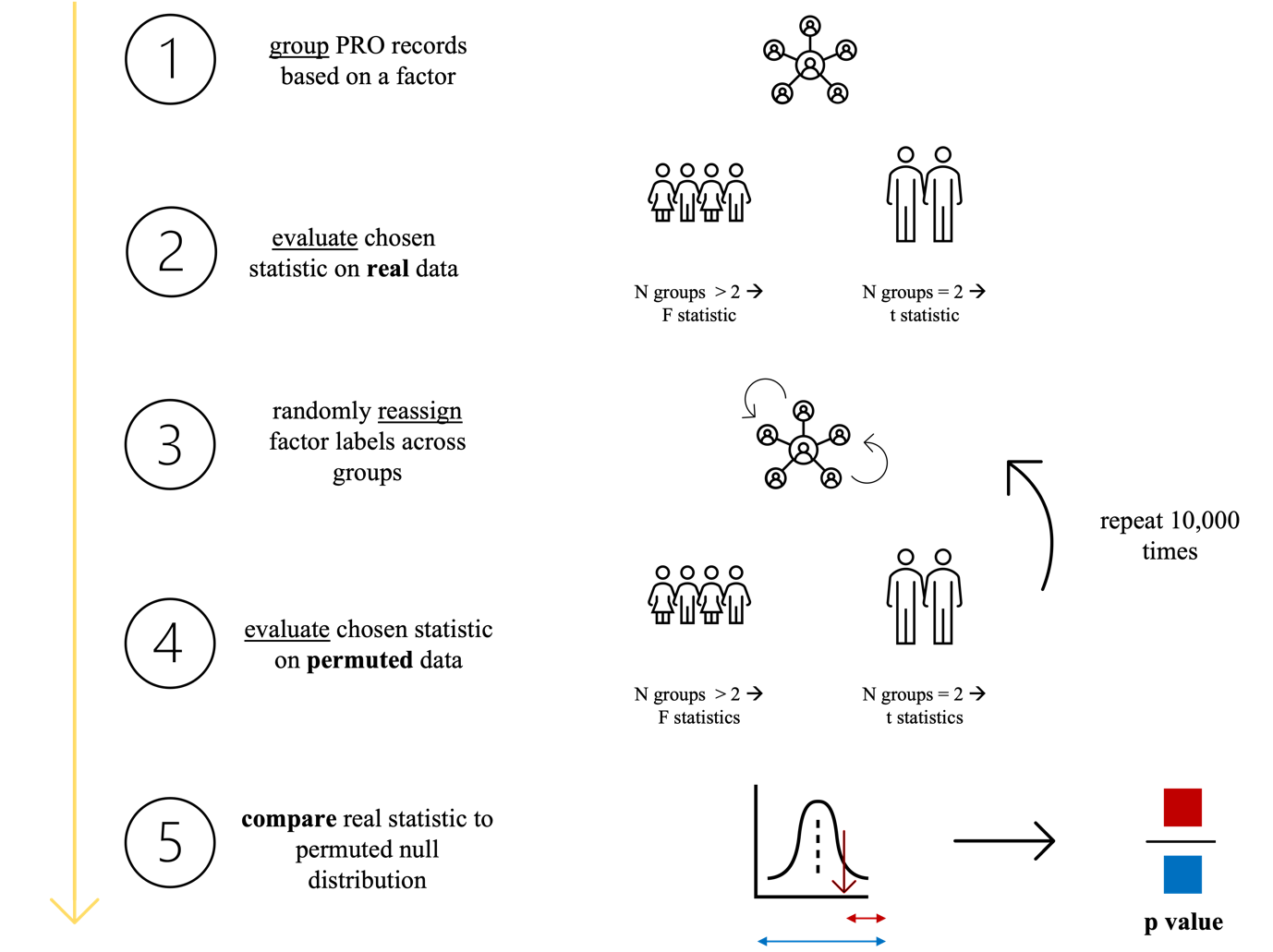


**Supplementary Figure 3 Null distributions of permuted F statistics and real F statistics located in red obtained evaluating the main effect of disease subtype and the interaction of subtype and duration on MSIS-29 motor and MSWS-12 using permutational multivariate analysis of variance with n=10,000 permutations.** The value of the real F statistic is reported in each plot. The resulting p value is indicated above each plot.


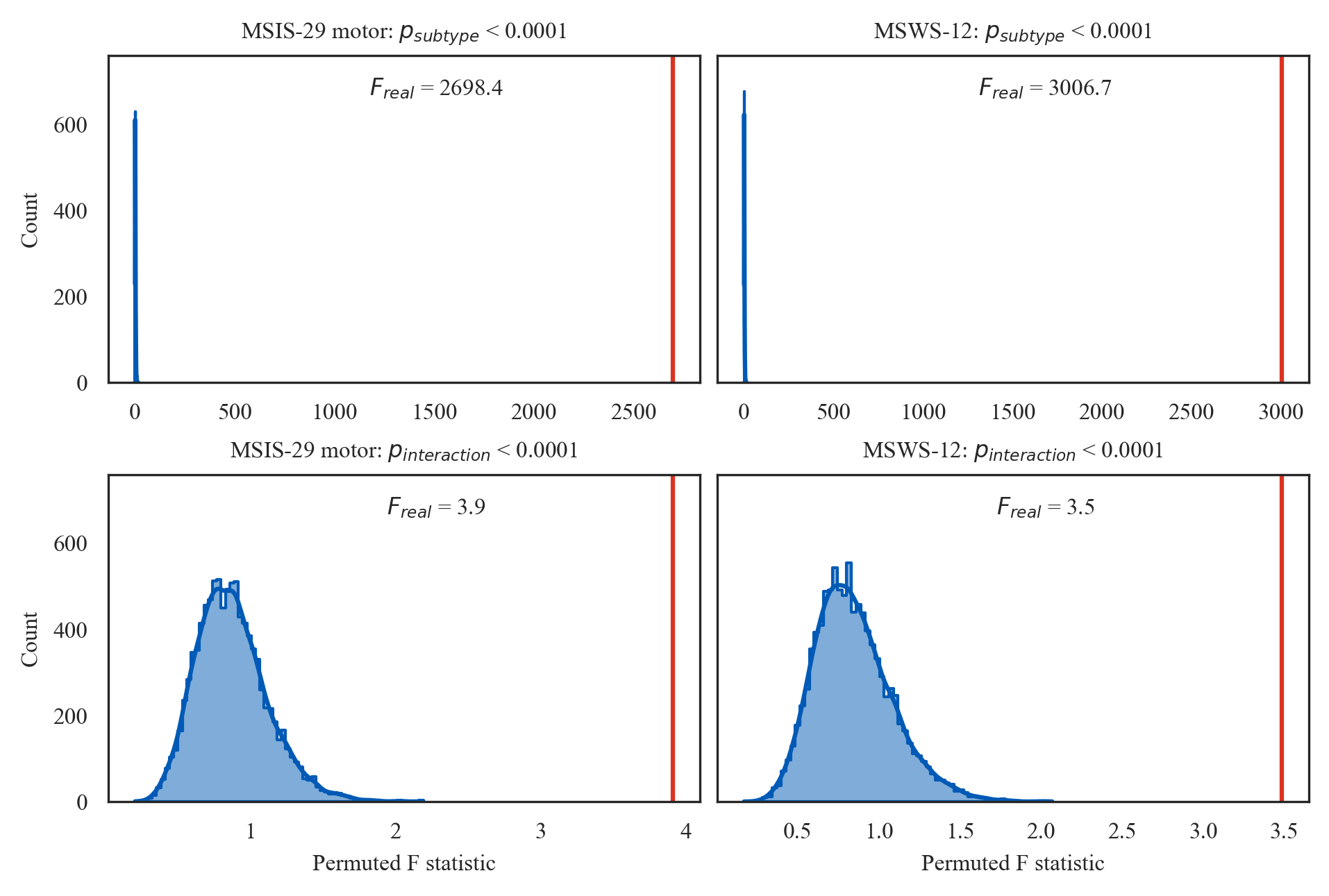


**Supplementary Figure 4 Null distributions of permuted F statistics and real F statistics located in red obtained evaluating the effect of disease duration on MSIS-29 motor and MSWS-12 separately for each subtype using permutational multivariate analysis of variance with n=10,000 permutations.** The value of the real F statistic is reported in each plot. The resulting p value is indicated above each plot.


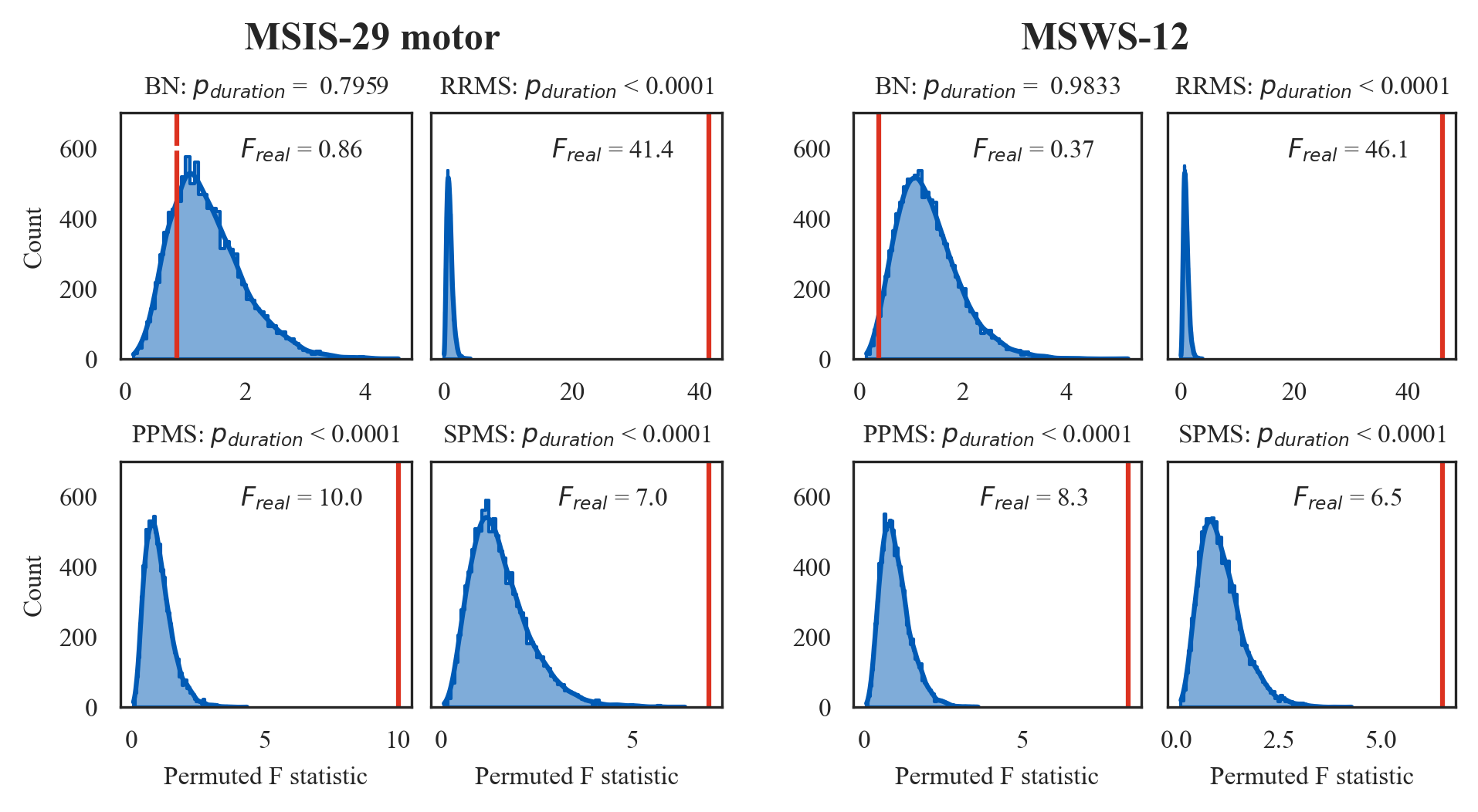


**Supplementary Figure 5 Null distributions of permuted F statistics and real F statistics located in red obtained evaluating the main effect of disease subtype on MSIS-29 motor and MSWS-12 separately across disease time bins using permutational multivariate analysis of variance with n=10,000 permutations.** The value of the real F statistic is reported in each plot. The resulting p value is indicated above each plot.


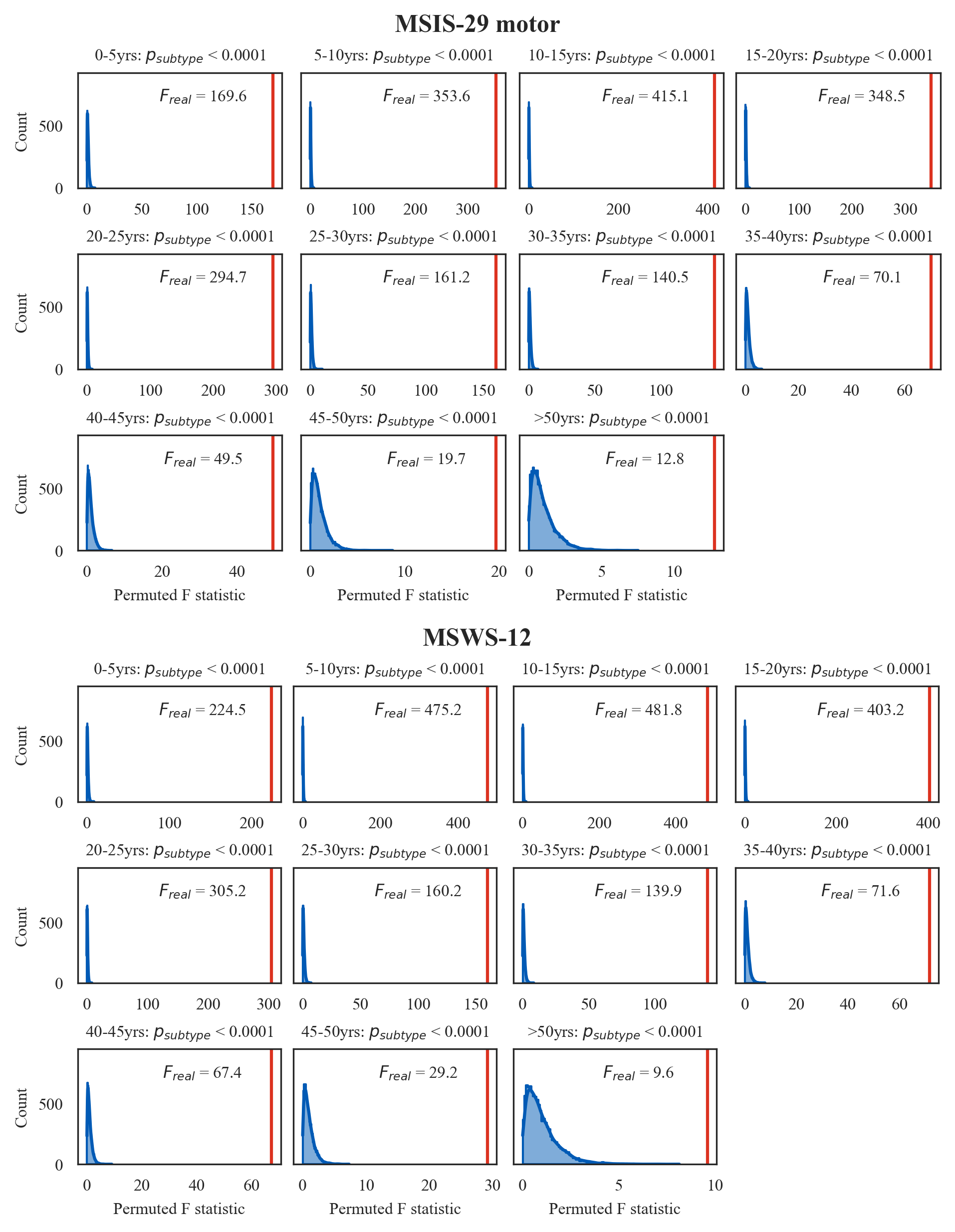


**Supplementary Figure 6 Null distributions of permuted t statistics and real t statistics located in red obtained comparing the MSIS-29 motor and MSWS-12 of relapsing individuals who do vs do not transition to progressive multiple sclerosis during the follow-up using permutation testing with t statistic and n=10,000 permutations.** The value of the real t statistic is reported in each plot. The resulting p value is indicated above each plot.

**
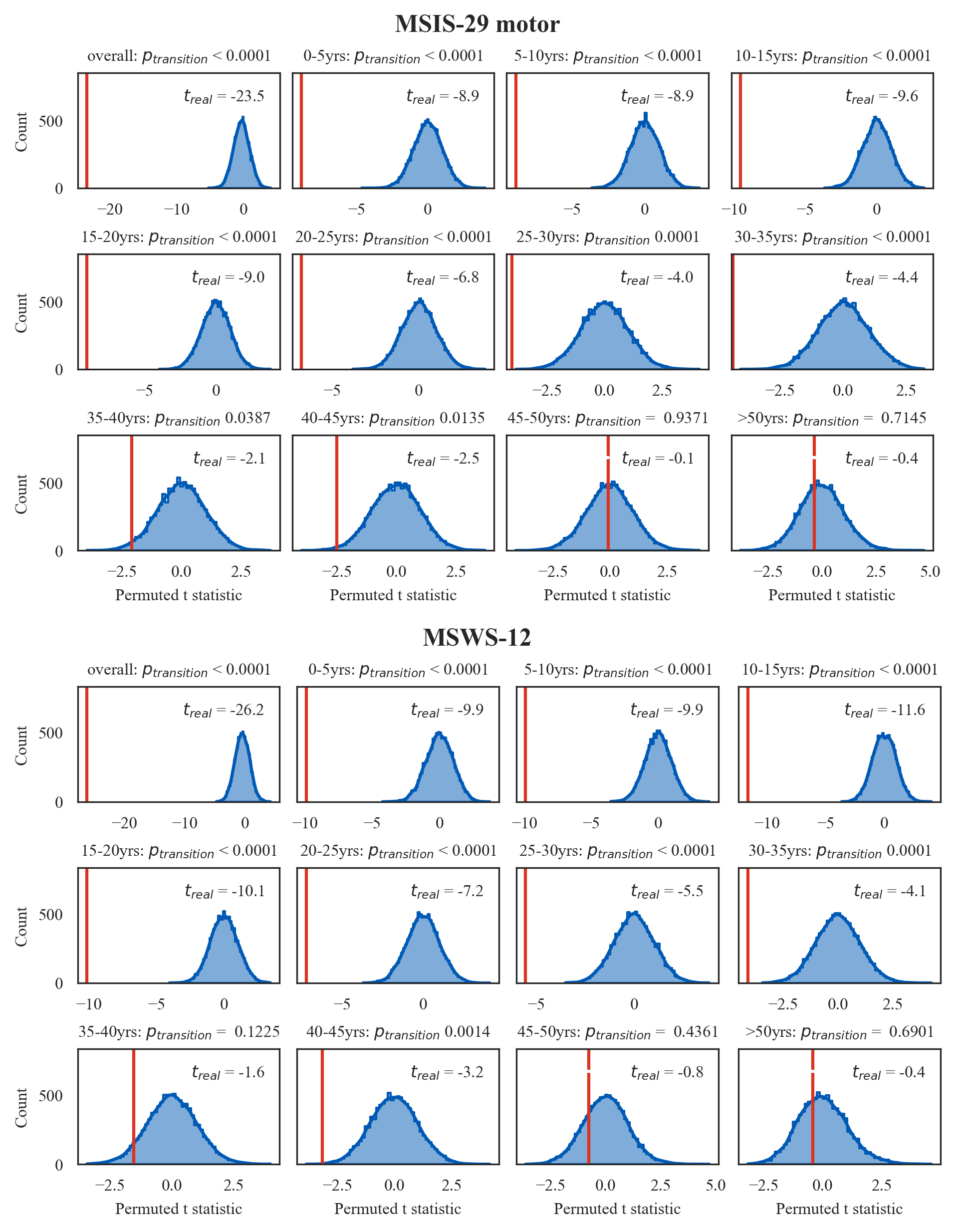
**

**Supplementary Table 1 Mean, standard deviation, and skew of the distributions of MSIS-29 motor and MSWS-12 across subtypes and disease time bins.**

|  |  |  | **Disease duration [yrs]** | | | | | | | | | | |
| --- | --- | --- | --- | --- | --- | --- | --- | --- | --- | --- | --- | --- | --- |
|  |  |  | **0-5** | **5-10** | **10-15** | **15-20** | **20-25** | **25-30** | **30-35** | **35-40** | **40-45** | **45-50** | **>50** |
| **MSIS-29** | **Mean** | **BN** | 36.9 | 39.3 | 32.6 | 36.8 | 35.0 | 34.9 | 33.8 | 33.1 | 33.2 | 36.1 | 33.3 |
|  |  | **RR** | 29.1 | 32.1 | 35.4 | 38.0 | 38.8 | 42.3 | 40.2 | 41.3 | 43.6 | 46.9 | 49.0 |
|  |  | **SP** | 52.4 | 57.6 | 61.5 | 62.9 | 62.8 | 62.2 | 61.7 | 61.0 | 62.3 | 60.8 | 60.2 |
|  |  | **PP** | 51.7 | 56.1 | 58.9 | 61.0 | 62.2 | 62.2 | 64.3 | 59.1 | 63.6 | 67.6 | 62.6 |
|  | **Standard deviation** | **BN** | 24.6 | 26.1 | 25.5 | 26.5 | 26.2 | 25.0 | 23.3 | 24.5 | 25.3 | 24.9 | 22.4 |
|  |  | **RR** | 22.5 | 23.5 | 24.0 | 24.3 | 23.7 | 24.1 | 22.6 | 23.2 | 22.5 | 24.0 | 25.0 |
|  |  | **SP** | 21.3 | 20.5 | 20.3 | 20.1 | 20.1 | 20.5 | 20.8 | 21.0 | 20.3 | 19.4 | 20.4 |
|  |  | **PP** | 23.6 | 22.9 | 21.7 | 21.0 | 21.0 | 20.9 | 19.5 | 23.6 | 25.5 | 19.9 | 12.4 |
|  | **Skew** | **BN** | 0.2 | 0.3 | 0.6 | 0.5 | 0.5 | 0.6 | 0.5 | 0.6 | 0.7 | 0.5 | 0.3 |
|  |  | **RR** | 0.8 | 0.6 | 0.4 | 0.3 | 0.3 | 0.2 | 0.4 | 0.2 | 0.1 | -0.1 | -0.1 |
|  |  | **SP** | -0.3 | -0.3 | -0.5 | -0.5 | -0.5 | -0.6 | -0.5 | -0.4 | -0.4 | -0.2 | -0.3 |
|  |  | **PP** | -0.0 | -0.2 | -0.4 | -0.4 | -0.4 | -0.6 | -0.7 | -0.4 | -0.6 | -0.9 | -0.4 |
| **MSWS-12** | **Mean** | **BN** | 35.0 | 37.9 | 33.9 | 35.5 | 36.4 | 37.4 | 34.3 | 33.1 | 32.3 | 34.6 | 40.9 |
|  |  | **RR** | 25.6 | 29.1 | 33.4 | 36.2 | 38.7 | 41.9 | 40.0 | 41.2 | 41.1 | 45.2 | 50.0 |
|  |  | **SP** | 57.9 | 63.1 | 67.0 | 68.3 | 68.8 | 67.7 | 67.5 | 66.6 | 68.8 | 67.6 | 65.5 |
|  |  | **PP** | 57.3 | 63.1 | 66.9 | 68.6 | 68.9 | 67.7 | 67.4 | 64.5 | 60.1 | 72.2 | 73.6 |
|  | **Standard deviation** | **BN** | 27.2 | 28.0 | 29.6 | 28.9 | 28.9 | 29.6 | 27.2 | 28.9 | 28.7 | 26.8 | 28.6 |
|  |  | **RR** | 24.3 | 25.7 | 26.4 | 26.5 | 26.4 | 26.5 | 24.8 | 26.3 | 23.1 | 26.0 | 24.4 |
|  |  | **SP** | 21.3 | 20.5 | 19.6 | 18.5 | 18.7 | 20.2 | 19.9 | 20.7 | 17.3 | 17.5 | 18.1 |
|  |  | **PP** | 24.1 | 21.0 | 19.9 | 19.6 | 18.6 | 19.2 | 21.3 | 25.4 | 26.0 | 17.9 | 13.5 |
|  | **Skew** | **BN** | 0.2 | 0.2 | 0.4 | 0.3 | 0.3 | 0.3 | 0.5 | 0.5 | 0.7 | 0.6 | 0.3 |
|  |  | **RR** | 0.8 | 0.6 | 0.4 | 0.2 | 0.1 | 0.0 | 0.2 | 0.0 | -0.1 | -0.1 | -0.3 |
|  |  | **SP** | -0.6 | -0.8 | -1.1 | -1.2 | -1.4 | -1.3 | -1.3 | -1.2 | -1.1 | -0.9 | -0.9 |
|  |  | **PP** | -0.6 | -0.9 | -1.2 | -1.4 | -1.2 | -1.0 | -1.1 | -1.2 | -1.0 | -1.3 | -0.6 |

**Supplementary Table 2 Real F statistics and p values obtained randomly reassigning the disease subtype labels of the MSIS-29 motor and MSWS-12 separately at each time bin.**

|  |  | **MSIS-29** | | **MSWS-12** | |
| --- | --- | --- | --- | --- | --- |
|  |  | **F_real_** | **p** | **F_real_** | **p** |
| **Disease durations [yrs]** | **0-5** | 169.6 | <0.0001 | 224.5 | <0.0001 |
|  | **5-10** | 354.6 | <0.0001 | 475.2 | <0.0001 |
|  | **10-15** | 415.1 | <0.0001 | 481.8 | <0.0001 |
|  | **15-20** | 348.5 | <0.0001 | 403.2 | <0.0001 |
|  | **20-25** | 294.7 | <0.0001 | 305.2 | <0.0001 |
|  | **25-30** | 161.2 | <0.0001 | 160.2 | <0.0001 |
|  | **30-35** | 140.5 | <0.0001 | 139.9 | <0.0001 |
|  | **35-40** | 70.1 | <0.0001 | 71.6 | <0.0001 |
|  | **40-45** | 49.5 | <0.0001 | 67.4 | <0.0001 |
|  | **45-50** | 19.7 | <0.0001 | 29.2 | <0.0001 |
|  | **>50** | 12.8 | <0.0001 | 9.6 | <0.0001 |

**Supplementary Table 3 Real t statistics and p values obtained from pairwise comparisons of the MSIS-29 motor and MSWS-12 of subtypes overall and at each disease time bin.**

|  |  |  | **MSIS-29** | | | | | | **MSWS-12** | | | | | |
| --- | --- | --- | --- | --- | --- | --- | --- | --- | --- | --- | --- | --- | --- | --- |
|  |  |  | **SP**  **RR** | **SP**  **BN** | **SP**  **PP** | **PP**  **RR** | **PP**  **BN** | **RR**  **BN** | **SP**  **RR** | **SP**  **BN** | **SP**  **PP** | **PP**  **RR** | **PP**  **BN** | **RR**  **BN** |
|  | **overall** | **t_real_** | 79.7 | 40.3 | 6.0 | 55.4 | 31.5 | -0.9 | 81.6 | 42.3 | 3.7 | 57.8 | 33.7 | -3.5 |
|  |  | **p** | <0.0001 | <0.0001 | <0.0001 | <0.0001 | <0.0001 | 0.5094 | <0.0001 | <0.0001 | 0.0213 | <0.0001 | <0.0001 | 0.0069 |
| **Disease durations [yrs]** | **0-5** | **t_real_** | 13.9 | 6.2 | 0.4 | 19.0 | 6.4 | -4.0 | 16.1 | 7.8 | 0.3 | 21.7 | 8.3 | -4.0 |
|  |  | **p** | <0.0001 | <0.0001 | 0.6947 | <0.0001 | <0.0001 | <0.0001 | <0.001 | <0.001 | 0.8043 | <0.0001 | <0.0001 | <0.001 |
|  | **5-10** | **t_real_** | 23.8 | 9.1 | 1.3 | 25.7 | 8.0 | -3.7 | 25.8 | 10.9 | 0.0 | 30.3 | 11.4 | -3.7 |
|  |  | **p** | <0.0001 | <0.0001 | 0.1925 | <0.0001 | <0.0001 | 0.0003 | <0.001 | <0.001 | 0.9833 | <0.0001 | <0.0001 | 0.0004 |
|  | **10-15** | **t_real_** | 28.7 | 16.1 | 2.5 | 25.0 | 13.8 | 1.5 | 29.2 | 15.7 | 0.1 | 27.7 | 15.3 | -0.2 |
|  |  | **p** | <0.0001 | <0.0001 | 0.0135 | <0.0001 | <0.0001 | 0.141 | <0.001 | <0.001 | 0.9426 | <0.0001 | <0.0001 | 0.817 |
|  | **15-20** | **t_real_** | 28.3 | 15.6 | 1.9 | 20.9 | 12.9 | 0.6 | 29.1 | 17.6 | -0.3 | 22.7 | 15.3 | 0.3 |
|  |  | **p** | <0.0001 | <0.0001 | 0.0671 | <0.0001 | <0.0001 | 0.5327 | <0.001 | <0.001 | 0.7893 | <0.0001 | <0.0001 | 0.755 |
|  | **20-25** | **t_real_** | 26.1 | 16.4 | 0.5 | 18.5 | 13.7 | 1.9 | 26.7 | 16.9 | -0.0 | 17.3 | 13.5 | 0.9 |
|  |  | **p** | <0.0001 | <0.0001 | 0.6388 | <0.0001 | <0.0001 | 0.0488 | <0.001 | <0.001 | 0.9735 | <0.0001 | <0.0001 | 0.3465 |
|  | **25-30** | **t_real_** | 18.5 | 14.5 | 0.0 | 11.9 | 11.8 | 3.3 | 19.5 | 13.6 | 0.0 | 11.0 | 9.9 | 1.6 |
|  |  | **p** | <0.0001 | <0.0001 | 0.9903 | <0.0001 | <0.0001 | 0.0012 | <0.001 | <0.001 | 0.9985 | <0.0001 | <0.0001 | 0.1118 |
|  | **30-35** | **t_real_** | 16.6 | 13.4 | -1.5 | 12.0 | 11.9 | 2.7 | 18.0 | 13.9 | 0.0 | 9.2 | 8.9 | 1.9 |
|  |  | **p** | <0.0001 | <0.0001 | 0.1456 | <0.0001 | <0.0001 | 0.0081 | <0.001 | <0.001 | 0.9723 | <0.0001 | <0.0001 | 0.0576 |
|  | **35-40** | **t_real_** | 11.7 | 10.7 | 0.8 | 6.3 | 7.0 | 2.6 | 12.6 | 10.7 | 0.7 | 5.6 | 5.9 | 2.0 |
|  |  | **p** | <0.0001 | <0.0001 | 0.4144 | <0.0001 | <0.0001 | 0.0088 | <0.001 | <0.001 | 0.5214 | <0.0001 | <0.0001 | 0.0458 |
|  | **40-45** | **t_real_** | 8.6 | 10.2 | -0.5 | 5.3 | 6.6 | 2.8 | 12.2 | 11.6 | 2.4 | 3.7 | 4.2 | 2.0 |
|  |  | **p** | <0.0001 | <0.0001 | 0.645 | <0.0001 | <0.0001 | 0.0055 | <0.0001 | <0.0001 | 0.0161 | 0.0003 | 0.0003 | 0.0492 |
|  | **45-50** | **t_real_** | 4.5 | 6.3 | -1.9 | 4.1 | 5.5 | 2.0 | 6.6 | 7.9 | -1.2 | 4.4 | 5.6 | 1.6 |
|  |  | **p** | <0.0001 | <0.0001 | 0.0664 | 0.0002 | <0.0001 | 0.0544 | <0.0001 | <0.0001 | 0.2513 | 0.0001 | <0.0001 | 0.1086 |
|  | **>50** | **t_real_** | 2.7 | 5.8 | -0.5 | 2.1 | 4.9 | 2.4 | 3.3 | 4.5 | -1.3 | 2.7 | 3.2 | 1.1 |
|  |  | **p** | 0.0071 | <0.0001 | 0.6325 | 0.0452 | 0.0001 | 0.018 | 0.0001 | <0.0001 | 0.1947 | 0.0084 | 0.0038 | 0.2778 |

**Supplementary Table 4 Real t statistics and p values obtained from pairwise comparisons of the MSIS-29 motor and MSWS-12 of relapsing individuals who do vs do not transition during the follow-up, at each disease time bin.**

|  |  | **MSIS-29** | | **MSWS-12** | |
| --- | --- | --- | --- | --- | --- |
|  |  | **t_real_** | **p** | **t_real_** | **p** |
| **Disease durations [yrs]** | **0-5** | -8.9 | <0.0001 | -9.9 | <0.0001 |
|  | **5-10** | -10.6 | <0.0001 | -11.9 | <0.0001 |
|  | **10-15** | -9.6 | <0.0001 | -11.6 | <0.0001 |
|  | **15-20** | -9.0 | <0.0001 | -10.1 | <0.0001 |
|  | **20-25** | -6.8 | <0.0001 | -7.2 | <0.0001 |
|  | **25-30** | -4.0 | 0.0001 | -5.5 | <0.0001 |
|  | **30-35** | -4.4 | <0.0001 | -4.1 | 0.0001 |
|  | **35-40** | -2.1 | 0.0387 | -1.6 | 0.1125 |
|  | **40-45** | -2.5 | 0.0135 | -3.2 | 0.0014 |
|  | **45-50** | -0.1 | 0.9371 | -0.8 | 0.4361 |
|  | **>50** | -0.4 | 0.7145 | -0.4 | 0.6901 |
